# Characteristics and Clinical Outcomes in Patients With Cirrhosis due to MASLD in Sweden

**DOI:** 10.1111/liv.70487

**Published:** 2025-12-26

**Authors:** Ying Shang, Linnea Widman, Xiao Zhang, Gail Fernandes, Matthew G. Melaragno, Samuel S. Engel, Johan Vessby, Mattias Ekstedt, Hannes Hagström

**Affiliations:** ^1^ Department of Medicine, Huddinge Karolinska Institutet Stockholm Sweden; ^2^ Merck & Co., Inc. Rahway New Jersey USA; ^3^ Department of Medical Sciences, Gastroenterology Research Group Uppsala University Uppsala Sweden; ^4^ Division of Internal Medicine, Department of Gastroenterology and Hepatology and Department of Health, Medicine, and Caring Sciences Linköping University Linköping Sweden; ^5^ Division of Hepatology, Department of Upper GI Karolinska University Hospital Stockholm Sweden

**Keywords:** cirrhosis, decompensated cirrhosis, MASLD

## Abstract

**Background and Aims:**

There is limited real‐world data on the prognosis of patients with cirrhosis due to metabolic dysfunction‐associated steatotic liver disease (MASLD). We sought to describe the characteristics and assess the clinical course of MASLD‐cirrhosis.

**Methods:**

A Swedish register‐based cohort including 2318 patients with compensated and 1339 with decompensated cirrhosis due to MASLD from 2001 to 2020 was analysed. Incidence rates of severe clinical events including decompensated cirrhosis, liver transplantation or overall mortality were estimated. We further compared the results using a clinical cohort of 293 patients with MASLD‐ cirrhosis from three University Hospitals.

**Results:**

Overall, 55.7% patients with compensated cirrhosis (median age 70, 43.6% female, 88.7% type 2 diabetes [T2D]) developed severe clinical events during a median follow‐up of 1.1 years. The incidence rate was 78.5/1000 person‐year (PY), 6.8/1000 PY and 182.0/1000 PY for decompensation, liver transplantation and death, respectively. Among 1399 patients with decompensated cirrhosis (median age 72, 41.1% female, 86% T2D), ascites (74.6%) was the most frequent initial events. 59.7% experienced death or transplantation with a median follow‐up of 0.58 years. The incidence rate was 14.0/1000 PY for liver transplantation and 353.2/1000 PY for overall death. Estimates were similar in the clinical cohort but lower overall mortality were observed (IR = 63.1/1000 PY in compensated cirrhosis and 188.2/1000 PY in decompensated events).

**Conclusions:**

This study describes the clinical characteristics and the natural history of patients with MASLD‐cirrhosis by compensation status in Sweden, providing estimates for important outcomes that may be useful for patient prognostication and clinical trial design.

## Lay Summary

1. The clinical course of patients with cirrhosis due to MASLD has been reported with mixed results, largely because of differing methodologies
2. Using Swedish national register data, we assessed the incidence rates of cirrhosis progression to decompensated cirrhosis, liver‐related death, and overall mortality, as well as the progression rates of decompensated cirrhosis.
3. The natural history data presented here may help inform clinical trial design by providing outcome rates from a large, nationwide sample with minimal loss to follow‐up or selection bias.

## 1 Background

Liver cirrhosis is the end‐stage of many chronic liver diseases and accounts for 2.4% of global deaths [1]. Cirrhosis is typically characterised into two states, compensated and decompensated cirrhosis; the latter usually defined by occurrence of ascites, hepatic encephalopathy, or variceal bleeding. The most common causes of cirrhosis in the Western countries, including Sweden, have been alcohol‐related liver disease and hepatitis C [2, 3]. However, the epidemiology of chronic liver disease has changed with less viral hepatitis and more metabolic dysfunction‐associated steatotic liver disease (MASLD) in recent years [1]. The rapidly increasing incidence of MASLD has led to a growing number of patients with cirrhosis due to this condition. Patients with cirrhosis due to MASLD tend to be older and have a higher burden of cardiometabolic comorbidities [4], and these factors elevate the risk of competing extrahepatic events, potentially altering the clinical course towards liver‐related outcomes and non‐liver death in an unpredictable manner.

There are limited real‐world data on the progression of patients with cirrhosis due to MASLD. Previous studies describing such outcomes mainly include patients across the full spectrum of MASLD, with only a small subset having cirrhosis [5, 6, 7, 8]. While some studies were conducted in patients with cirrhosis, they often studied various etiologies rather than MASLD specifically [4, 9, 10]. The current knowledge of progression in cirrhosis caused by MASLD is largely based on data from specialised care settings of patients with biopsy‐proven diagnosis. These studies typically have smaller sample sizes and may be vulnerable to selection bias due to the biopsy requirement, which is not commonly performed in cirrhosis in most clinical settings. The estimates are mixed due to different referral patterns and levels of healthcare. For instance, data from a multi‐national study of 299 patients with biopsy‐confirmed MASLD with compensated cirrhosis found that 44% progressed to decompensated cirrhosis within a median follow‐up of 5.5 years [8]. In contrast, the US NASH Clinical Research Network study of patients with biopsy‐proven NAFLD found that 11.1% (17/153) of patients with F4 progressed to decompensated events during a median of 4 years [7].

Understanding the characteristics of patients with cirrhosis due to MASLD is crucial for effective management and clinical trial design. Identifying factors associated with a high risk of progression to severe clinical events is important for risk stratification and improving patient outcomes, which often requires detailed laboratory data. In this study, we aim to (1) describe the characteristics and long‐term outcomes of patients with cirrhosis due to MASLD using a large nationwide register, and (2) validate the register‐based results and identify prognostic factors for progression using a large Swedish clinical cirrhosis cohort with detailed laboratory data. These complementary approaches may offer a comprehensive view of the clinical course of MASLD cirrhosis.

## 2 Methods

### 2.1 The Register‐Based Cohort

The first part of this study used data from the nationwide “DEcoding the epidemiology of LIVER disease in Sweden” (DELIVER) cohort, including all patients with administrative codes of chronic liver disease from the National Patient Register (NPR) between 1964 and 2020 [11]. The NPR initially covered only inpatient care until 2001, at which point it also expanded to all outpatient visits from the specialised care [12]. To provide a more contemporary description of the clinical course, we used data from 2001 to 2020 to identify patients with MASLD cirrhosis from both inpatient and outpatient care.

Cirrhosis, including compensated and decompensated cirrhosis, was identified based on ICD codes (Table S1). Decompensated events included ascites, hepatic encephalopathy, and oesophageal variceal bleeding. Cirrhosis due to MASLD was ascertained if patients received a MASLD diagnosis either prior to or within three months following cirrhosis diagnosis, without the presence of other competing etiologies of cirrhosis. In cases where MASLD was not explicitly diagnosed, the presence of T2D or obesity before or within three months after cirrhosis diagnosis was used as a proxy to cirrhosis due to MASLD, in line with a previous expert consensus report [13]. If the patients received the diagnosis of MASLD or T2D or obesity prior to the diagnosis of cirrhosis, the first date when cirrhosis was reported was used as the index date. If the diagnosis of MASLD or T2D or obesity occurred after the diagnosis of cirrhosis, the date of diagnosis of MASLD or T2D or obesity was used as the index date to minimise immortal time bias, as the patients would have survived to the diagnosis of MASLD or T2D or obesity following cirrhosis diagnosis.

**FIGURE 1:**
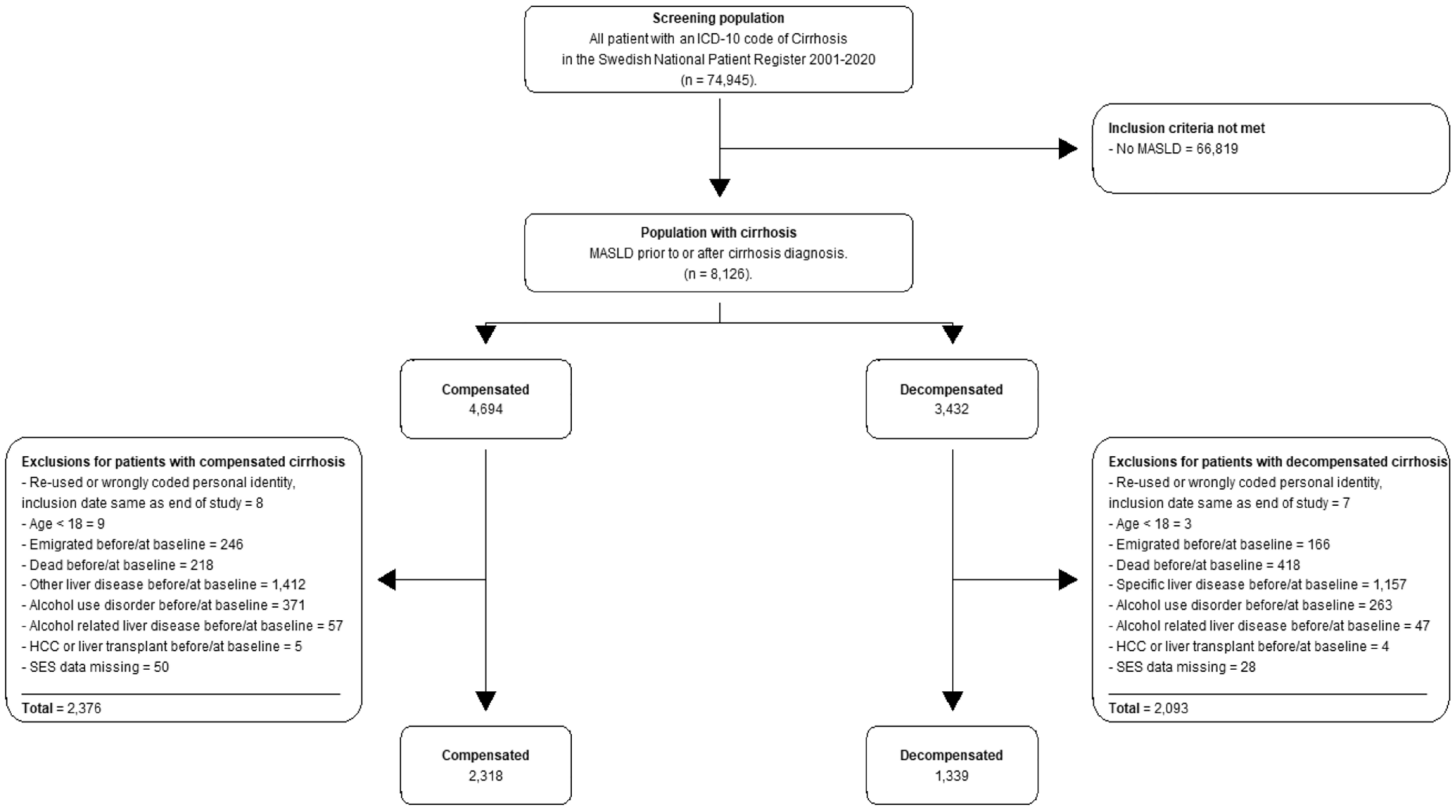
Flowchart of the study population.

We excluded patients with the following conditions prior to or at the index date: age < 18 years, emigration or death, other etiologies of liver disease rather than MASLD, alcohol use disorder or alcohol‐related liver disease, HCC or liver transplantation, missing socioeconomic status, or those with administrative miscoding, leaving 2318 patients with compensated cirrhosis and 1339 with decompensated events (Figure 1). Patients who obtained a diagnosis of a competing liver disease other than MASLD, or alcohol use disorder during follow‐up were censored at that time. The NPR has been externally validated with a positive predictive value ranging from 85% to 95%, depending on the diseases [12]. Notably, the positive predictive value of diagnoses corresponding to MASLD after exclusion of coding with other liver diseases and was 91%–95% for cirrhosis or decompensated cirrhosis [14, 15].

### 2.2 Outcomes

We used data from the NPR, the Swedish Cancer Register, and the Cause of Death Register to identify outcomes between 2001 and 2020 [12, 16, 17]. For patients with compensated cirrhosis, the primary outcome was severe clinical events, a composite endpoint including fatal or non‐fatal hepatic decompensated events (i.e., ascites, encephalopathy, or variceal bleeding); liver transplantation; and death from any cause, which is in accordance with the definition of outcomes in a cirrhotic population defined by the Food and Drug Administration when performing clinical trials (available at: https://www.fda.gov/regulatory‐information/search‐fda‐guidance‐documents/nonalcoholic‐steatohepatitis‐compensated‐cirrhosis‐developing‐drugs‐treatment‐guidance‐industry), although progression to MELD scores of 15 or more could not be captured due to the lack of laboratory data in the used registers. For patients with decompensated cirrhosis at baseline, the primary outcome included liver transplantation and death from any cause. Secondary outcomes were individual components of severe clinical events (e.g., ascites) in patients with compensated cirrhosis and hepatocellular carcinoma (HCC) as well as liver‐related mortality (recorded as the primary or a contributing cause of death from the Causes of Death Register in patients with compensated or decompensated cirrhosis). Both primary and secondary endpoints were recorded at the first event, and the recurrence of the same event or a new event belonging to the same category was not included. All outcomes were identified using ICD‐10 codes (Table S1).

### 2.3 Covariates

Information on the history of comorbidities prior to or at the index date was identified from the NPR and the Swedish Cancer Register. These comorbidities included cardiometabolic diseases such as type 2 diabetes, cardiovascular disease, obesity, hypertension, or hyperlipidemia, and other common health conditions such as chronic obstructive pulmonary disease, renal impairment, hypothyroidism, and extrahepatic cancer (ICD‐10 codes in Table S1). Information on the medication prescription before or within 30 days of index date was identified using Anatomical Therapeutic Chemical codes from the National Prescribed Drug Register. Established in July 2005, this register includes records of all prescribed drugs dispensed from pharmacies in Sweden [18].

### 2.4 The Clinical Cirrhosis Cohort

To explore factors affecting progression in patients with MASLD cirrhosis, a clinical cohort of 2679 patients with cirrhosis of any aetiology with detailed laboratory tests from three Swedish University hospitals from 1980 to 2020 was used [10, 19]. The diagnosis of cirrhosis was confirmed and defined if a patient had a liver biopsy showing fibrosis stage 4, vibration‐controlled transient elastography with at least 15 kPa, a radiological finding of cirrhosis (e.g., presence of portal hypertension, shrunken nodular liver without other competing cause), or clinical diagnosis based on manifestations or laboratory findings (i.e., oesophageal varices, ascites, or hepatic encephalopathy). The diagnosis of MASLD was based on histology (at least stage 1 steatosis), radiology, VCTE with CAP or a clinical diagnosis, described in the previous publication [10, 19]. We excluded patients with cirrhosis due to other etiologies than MASLD, HCC or liver transplantation at baseline, or reused personal identity numbers, which left 225 patients with compensated and 68 with decompensated cirrhosis due to MASLD (Figure S1). Body mass index and lab values were obtained within a month of diagnosis and scoring systems indicating disease severity including the Model for End‐Stage Liver Disease (MELD), Fibrosis‐4 (FIB‐4) and Child‐Turcotte‐Pugh (CTP) were calculated. FIB‐4 was categorised as low (FIB‐4 < 1.3 for patients ≤ 65, < 2.0 for patients > 65 years), intermediate (< 2.67), and high risk for fibrosis (≥ 2.67). CTP was categorised into A (score of 5–6), B (7–9) and C (10–15). The cohort was followed up until December 31st, 2020. Severe clinical events were defined in the same manner as the events for the register‐based cohort but were defined from chart reviews. Register linkage was not conducted in this cohort.

### 2.5 Statistical Analysis

In both cohorts, the baseline characteristics were described using medians with interquartile range (IQR), or as the total numbers with percentages where applicable. The incidence rates (IR, per 1000 person‐years [PY]) of the outcomes were calculated as the number of events developed during follow‐up divided by the number of person‐years at risk. Follow‐up time was calculated from index date until the outcome occurred, emigration, diagnosis of specific liver diseases rather than MASLD, or end of the study period (December 31st, 2020), whichever came first. Incidence rates for secondary outcomes were calculated for patients with compensated and decompensated cirrhosis separately.

Cumulative incidence of severe clinical events was calculated at 1, 5, and 10 years. In the register‐based cohort, cumulative incidences for the secondary outcomes were calculated accounting for the competing risk of death due to non‐liver events (e.g., non‐liver related death was considered a competing event to decompensated cirrhosis). Since patients may be hospitalised for other conditions that confer a high mortality rate, for instance an infection with cirrhosis found accidentally during this hospitalisation event, the analyses were repeated including only patients diagnosed in the outpatient setting, by using only data from the outpatient part of the National Patient Register. In addition, the cumulative incidence was estimated after excluding patients with compensated cirrhosis who developed severe clinical events within the first year, hence starting follow‐up one year after the initial index date. This was done to reduce the risk of detection bias. All analyses were conducted by STATA 16 and 17 (Stata Corp, College Station, TX).

### 2.6 Additional Analysis in the Clinical Cirrhosis Cohort

As granular lab data including MELD and CTP indicating the severity of cirrhosis were available in the cirrhosis cohort, we investigated the clinical factors associated with progression in cirrhosis. Hazard ratios (HR) were estimated from Cox regression proportional hazard models to identify factors associated with incident severe clinical events in patients with compensated and decompensated cirrhosis, respectively. The models were adjusted for age, sex, calendar year of diagnosis, body mass index and the presence of type 2 diabetes. The models were additionally adjusted for each scoring system (i.e., FIB‐4, CTP, MELD) separately to identify factors associated with higher progression rates. A two‐sided *p*‐value < 0.05 was considered statistically significant. The study and data collection process were approved by the Regional Ethical Review Board of Stockholm (dnr 2018/880–31, dnr 2016/1772‐31 and dnr 2018/450 for the clinical cohort; dnr 2017/1019‐31/1 for the register cohort).

## 3 Results

### 3.1 Characteristics of Patients With MASLD Cirrhosis in Both Cohorts

In the register cohort, among 2318 patients diagnosed with compensated cirrhosis due to MASLD, the median age was 70 (63–77) years, and 43.6% were female (Table 1). Cardiometabolic diseases were common, with 88.7% patients having formal diagnoses of type 2 diabetes, 81.0% of hypertension, 54.0% of hyperlipidemia, 22.0% of obesity and 35.1% of established cardiovascular disease. 23.5% had a previous history of depression or anxiety, and 33.0% had any previous extrahepatic cancer.

**TABLE 1:** Baseline characteristics of patients with cirrhosis due to MASLD (presented as *n* (%) or median (IQR)) from the register‐based cohort.

	All	Compensated cirrhosis	Decompensated cirrhosis
No. of individuals	3657	2318	1339
*Demographic*			
Age at diagnosis	71 (63–78)	70 (63–77)	72 (64–78)
Female	1561 (42.7)	1011 (43.6)	550 (41.1)
*Comorbidity*			
Ascites	999 (27.3)	0 (0.0)	999 (74.6)
Varices bleeding	374 (10.2)	0 (0.0)	374 (27.9)
Hepatic encephalopathy	14 (0.4)	0 (0.0)	14 (1.0)
Type 2 diabetes	3208 (87.7)	2057 (88.7)	1151 (86.0)
Cardiovascular disease	1288 (35.2)	814 (35.1)	474 (35.4)
Obesity	744 (20.3)	510 (22.0)	234 (17.5)
Hypertension	2923 (79.9)	1877 (81.0)	1046 (78.1)
Hyperlipidemia	1956 (53.5)	1252 (54.0)	704 (52.6)
Chronic obstructive pulmonary disease	333 (9.1)	215 (9.3)	118 (8.8)
Depression or anxiety	833 (22.8)	544 (23.5)	289 (21.6)
Renal impairment	338 (9.2)	200 (8.6)	138 (10.3)
Hypothyroidism	335 (9.2)	199 (8.6)	136 (10.2)
Extrahepatic cancer	1260 (34.5)	765 (33.0)	495 (37.0)
*Medications use*			
Any Antidiabetics	1178 (32.2)	798 (34.4)	380 (28.4)
Metformin	406 (11.1)	304 (13.1)	102 (7.6)
GLP‐1 RA	78 (2.1)	69 (3.0)	9 (0.7)
TZD	3 (0.1)	2 (0.1)	1 (0.1)
SGLT‐2 inhibitors	42 (1.1)	34 (1.5)	8 (0.6)
Insulin	697 (19.1)	459 (19.8)	238 (17.8)
Any antihypertensive	1048 (28.7)	724 (31.2)	324 (24.2)
ACE	603 (16.5)	427 (18.4)	176 (13.1)
Beta blocker	547 (15.0)	363 (15.7)	184 (13.7)
Diuretics	41 (1.1)	33 (1.4)	8 (0.6)
Calcium blocker	289 (7.9)	215 (9.3)	74 (5.5)
SSRIs	121 (3.3)	81 (3.5)	40 (3.0)
Other antidepressants	65 (1.8)	42 (1.8)	23 (1.7)
Statins	461 (12.6)	328 (14.2)	133 (9.9)
Other antihyperlipidemic drugs	25 (0.7)	17 (0.7)	8 (0.6)
Vitamin E	4 (0.1)	2 (0.1)	2 (0.1)

Abbreviations: ACE, angiotensin‐converting enzyme; GLP‐1 RA, glucagon‐like peptide‐1 receptor agonist; SGLT‐2, sodium‐glucose transport protein 2; SSRI, selective serotonin reuptake inhibitors; TZD, thiazolidinedione.

In 1339 patients with decompensated cirrhosis, the median age at diagnosis was 72 (IQR 64–78) years, and 41% were female. The most frequent prior decompensated event was ascites (74.6%), followed by oesophageal variceal bleeding (27.9%) and hepatic encephalopathy (1.0%), with 5.8% having more than one prior decompensated event. The most common comorbidities were type 2 diabetes (86.0%), hypertension (78.1%), hyperlipidemia (52.6%), and extrahepatic cancer (37.0%).

Patients were generally younger in the clinical cohort than in the register cohort; the median age for patients diagnosed with compensated was 67 (IQR 60–73), and for decompensated events was 68 (IQR 61–74) (Table S2). 65.3% of patients with compensation and 79.4% with decompensated cirrhosis had type 2 diabetes. The median MELD was 9 (IQR 7–10) for patients with compensated cirrhosis and 11 for those with decompensated events. Furthermore, in 68 patients with decompensated cirrhosis, 77.8% had prior ascites and 27.9% had variceal bleeding.

### 3.2 Progression to Severe Clinical Events in Patients With Compensated Cirrhosis in the Register‐Based Cohort

During a median follow‐up of 1.1 years (IQR 0.22–3.27, range 0.0–20.0), 1290 of the 2328 (55.6%) patients with compensated cirrhosis at baseline developed severe clinical events (IR 243.3/1000 PY, 95% CI 230.4–256.8) (Table 2). Of these, 18% (78.5/1000 PY) developed decompensated cirrhosis, 1.7% (6.8/1000 PY) underwent liver transplantation, and 47.3% (182.3/1000 PY) died during follow‐up. The most frequent incident decompensated event was ascites (10.9%, 44.4/1000 PY) followed by variceal bleeding (7.8%, 31.4/1000 PY). Incidence rates of the outcomes after excluding first year of the outcomes were shown in Supplementary Table 5.

**TABLE 2:** Clinical outcomes in patients with cirrhosis due to MASLD from the register‐based cohort.

Outcomes	Patients with compensated cirrhosis			Patients with decompensated cirrhosis		
	No. outcomes/no. patients	Follow‐up years (median, IQR)	IR (per 1000 py)	No. outcomes/no. patients	Follow‐up years (median, IQR)	IR (per 1000 py)
Severe clinical events	1290/2318 (55.7%)	1.10 (0.22–3.27)	243.3 (230.4–256.8)	799/1339 (59.7%)	0.58 (0.13–2.01)	385.2 (359.4–412.9)
Decompensated cirrhosis	422/2318 (18.2%)	1.14 (0.22–3.35)	78.5 (71.4–86.4)	—	—	—
Ascites	252/2318 (10.9%)	1.30 (0.24–3.60)	44.4 (39.2–50.2)	—	—	—
Encephalopathy	63/2318 (2.7%)	1.45 (0.29–3.72)	10.6 (8.3–13.6)	—	—	—
Variceal bleeding	179/2318 (7.7%)	1.31 (0.27–3.57)	31.4 (27.1–36.4)	—	—	—
Liver transplantation	40/2318 (1.7%)	1.45 (0.29–3.72)	6.80 (4.99–9.27)	29/1339 (2.2%)	0.58 (0.13–2.01)	14.0 (9.72–20.12)
All‐cause mortality	1096/2318 (47.3%)	1.50 (0.30–3.84)	182.3 (171.9–193.5)	777/1339 (58.0%)	0.60 (0.13–2.09)	353.2 (329.2–378.9)
Liver‐related mortality	165/2318 (7.1%)	1.50 (0.30–3.84)	27.4 (23.6–32.0)	156/1339 (11.7%)	0.60 (0.13–2.09)	70.9 (60.6–83.0)
HCC	291/2318 (12.6%)	1.26 (0.23–3.57)	51.5 (45.9–57.7)	152/1339 (11.4%)	0.55 (0.11–2.00)	73.1 (62.3–85.7)

Abbreviations: HCC, hepatocellular carcinoma; IR, incidence rate; MALO, major adverse liver outcomes; MASLD, metabolic dysfunction‐associated steatotic liver disease; py, person‐year.

**FIGURE 2:**
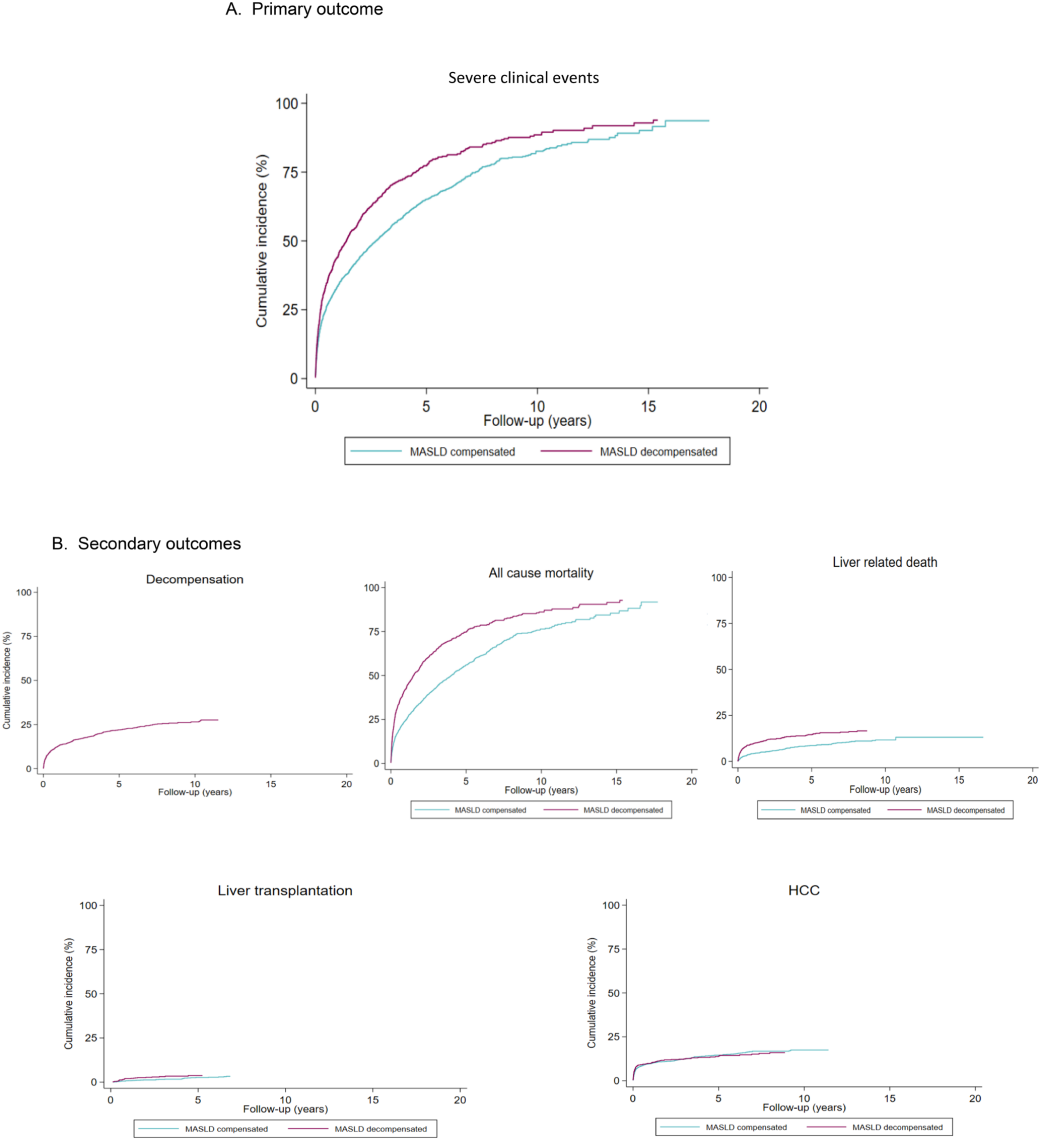
Cumulative incidence of severe clinical events (A) and individual events (B) in patients with compensated cirrhosis due to MASLD, and death, liver‐related death, liver transplant, and hepatocellular carcinoma in patients with decompensated cirrhosis. (A) Primary outcome. (B) Secondary outcomes.

The cumulative incidence of decompensated cirrhosis, liver transplantation or death in patients with compensated cirrhosis was 33.5% after 1 year, 65.1% after 5 years and 82.0% after 10 years (Table 3 and Figure 2A). This was largely driven by overall mortality, reaching 24.9% after 1 year, 56.0% after 5 years, and 76.1% after 10 years (Table 3 and Figure 2B). Liver‐specific mortality was 4.3% at 1 year, 8.5% at 5 years, and 11.3% at 10 years when adjusted for competing risks from non‐liver related death. The cumulative incidences of these outcomes were not materially changed among patients identified from the outpatient care only (Table S4).

**TABLE 3:** Cumulative incidence (%) and 95% confidence interval of the clinical outcomes in patients with cirrhosis due to MASLD from the register‐based cohort.

	1 year	5 years	10 years	Full follow‐up
*Patients with compensated cirrhosis*				
Severe clinical events	33.5 (31.5–35.5)	65.1 (62.5–67.5)	82.0 (79.1–84.5)	93.6 (86.8–97.0)
Decompensated cirrhosis	12.8 (11.4–14.3)	21.9 (19.9–23.9)	26.5 (24.0–29.0)	27.6 (24.8–30.4)
Liver transplantation	0.8 (0.5–1.3)	2.5 (1.8–3.5)	—	3.3 (2.3–4.5)
All‐cause mortality	24.6 (22.8–26.5)	56.0 (53.3–58.5)	76.1 (72.8–79.0)	91.7 (84.2–95.8)
Liver‐related mortality	4.3 (3.4–5.2)	8.5 (7.2–10.0)	11.3 (9.6–13.3)	13.2 (10.0–16.8)
HCC	9.7 (8.4–11.0)	14.6 (13.0–16.3)	17.1 (15.1–19.2)	17.5 (15.4–19.8)
*Patients with decompensated cirrhosis*				
Severe clinical events	44.0 (41.1–46.9)	77.3 (73.9–80.3)	88.0 (84.5–90.8)	93.9 (89.1–96.6)
Liver transplantation	2.0 (1.3–3.1)	3.7 (2.5–5.4)	—	3.7 (2.5–5.4)
All‐cause mortality	42.4 (39.5–45.2)	74.6 (71.2–77.7)	85.6 (81.9–88.6)	92.7 (87.5–95.8)
Liver‐related mortality	9.4 (7.9–11.2)	14.6 (12.4–16.9)	—	16.6 (14.0–19.4)
HCC	9.9 (8.3–11.7)	13.8 (11.7–16.1)	—	16.0 (13.4–18.9)

Abbreviations: HCC, hepatocellular carcinoma; MASLD, metabolic dysfunction‐associated steatotic liver disease.

Table 4 details the distribution of events during the first year, and in the years after. During the first year, 13.5% of patients developed any decompensated events, 24.9% died, and 9.4% developed HCC. Endocrinological diseases and extrahepatic cancers accounted for 9.3% and 9.0% of all deaths during the first year, while liver diseases only accounted for 4.3%. After the first year, event rates were generally lower.

**TABLE 4:** Number and proportion of events by follow‐up time in patients with MASLD‐ cirrhosis from the register‐based cohort.

	Year 1	Year 2 and after
*Patients with compensated cirrhosis*		
Deaths	577 (24.9%)	837 (36.1%)
By cause		
Liver disease	100 (4.3%)	133 (5.7%)
Extrahepatic cancer	208 (9.0%)	190 (8.2%)
Cardiovascular disease	119 (5.1%)	188 (8.1%)
Respiratory disease	86 (3.7%)	150 (6.5%)
Endocrine	216 (9.3%)	376 (16.2%)
Injury	25 (1.1%)	41 (1.8%)
Other	98 (4.2%)	161 (6.9%)
Decompensated events	314 (13.5%)	238 (10.3%)
Ascites	179 (7.7%)	154 (6.6%)
Variceal haemorrhage	124 (5.3%)	100 (4.3%)
Hepatic encephalopathy	30 (1.3%)	70 (3.0%)
Liver transplantation	22 (0.9%)	39 (1.7%)
HCC	217 (9.4%)	128 (5.5%)
*Patients with decompensated cirrhosis*		
Deaths	558 (41.7%)	416 (31.1%)
By cause		
Liver disease	125 (9.3%)	66 (4.9%)
Extrahepatic cancer	181 (13.5%)	77 (5.8%)
Cardiovascular disease	97 (7.2%)	71 (5.3%)
Respiratory disease	77 (5.8%)	75 (5.6%)
Endocrine	185 (13.8%)	168 (12.5%)
Injury	18 (1.3%)	17 (1.3%)
Other	102 (7.6%)	90 (6.7%)
Liver transplantation	24 (1.8%)	19 (1.4%)
HCC	127 (9.5%)	51 (3.8%)

Abbreviations: HCC, hepatocellular carcinoma; MASLD, metabolic dysfunction‐associated steatotic liver disease.

### 3.3 Progression to Liver Transplantation and Death in Patients With Decompensated Cirrhosis in the Register‐Based Cohort

Among 1339 patients with decompensated events, 799 (59.7%, IR 385.2/1000 PY, 95% CI = 359.4–412.9) developed severe clinical events during a median follow‐up of 0.58 years (IQR: 0.13–2.01, range 0.003–18.94), including 777 (58.0%, IR: 353.2/1000 PY) deaths and 29 (2.1%, IR: 14.0/1000 PY) liver transplantations. The cumulative incidence of death or transplant was 44.0%, 77.3%, and 88.0% over 1 year, 5 years, and 10 years, respectively, with overall mortality comprising the largest proportion, though liver‐related mortality was lower (Table 3). The high mortality rate during the first year in patients with decompensated cirrhosis was mainly attributed to endocrinological disease (13.9%), extrahepatic cancer (13.8%) and liver‐related death (9.3%). 9.5% of patients were diagnosed with HCC during the first year.

### 3.4 Clinical Cirrhosis Cohort: Progression Rates and Prognostic Factors

Among 225 patients with compensated cirrhosis, 50.2% (IR 104.5/1000 PY, 95% CI 86.9–125.7) patients developed severe clinical events during a median follow‐up of 3.8 years (Table S3). Decompensated events occurred in 34.7% (IR 71.5/1000 PY, 95% CI 57.3–89.3), liver transplantation occurred in 5.3% (IR 10.1/1000 PY, 95% CI 5.9–17.4), and death occurred in 38.2% (IR 63.1/1000 PY, 95% CI 51.5–77.9). Liver‐related deaths comprised 37.2% (32 out of 86) of the total mortality. In 68 patients with decompensated cirrhosis, 73.5% (IR 393.2, 95% CI 298.0–518.8) experienced the outcomes, with 25% (IR 133.7/1000 PY, 95% CI 83.1–215.1) undergoing liver transplantation and 54.4% (IR 188.2/1000 PY, 95% CI 136.4–259.7) dying. Liver‐related death accounted for 59.5% (22/37) of overall death.

In multivariable models, progression to severe clinical events in patients with compensated cirrhosis was associated with type 2 diabetes (adjusted HR 1.83, 95% CI 1.18–2.84) and higher MELD scores (adjusted HR 1.16, 95% CI 1.11–1.22 per one unit increase). Compared to CTP A, CTP B (adjusted HR: 2.99, 95% CI 1.81–4.93) and CTP C (adjusted HR: 23.9, 95% CI 5.18–110.0) were also associated with a higher risk of severe clinical events, although there were only two patients with CTP C at baseline (Table 5). When examining liver‐related events and non–liver‐related mortality separately, type 2 diabetes was associated only with liver disease progression, indicating that it is a driver of hepatic deterioration rather than non‐liver‐related mortality in MASLD cirrhosis (Table S6). In patients with decompensated cirrhosis, higher MELD and CTP scores were associated with faster progression to severe clinical events.

**TABLE 5:** Clinical factors associated with severe clinical events in patients with compensated cirrhosis or decompensated cirrhosis from the clinical cirrhosis cohort.

Patients with compensated cirrhosis															
	Crude HR	95% CI	*p*	HR^a^	95% CI	*p*	HR^b^	95% CI	*p*	HR^c^	95% CI	*p*	HR^d^	95% CI	*p*
Body mass index	0.96	0.93–0.99	0.034	0.97	0.94–1.00	0.087	0.97	1.06–2.55	0.138	0.97	0.93–1.00	0.067	0.98	0.94–1.01	0.244
Type 2 diabetes	1.70	1.15–2.58	0.012	1.83	1.18–2.84	0.007	1.76	1.12–2.74	< 0.001	1.81	1.17–2.81	0.008	1.75	1.12–2.73	0.013
AST, U/l	1.00	0.99–1.01	0.197												
ALT, U/l	1.00	1.00–1.01	0.736												
Bilirubin, mg/dl	1.11	1.05–1.18	< 0.001												
Total platelet counts, 10^9^	0.99	0.99–0.99	0.001												
Albumin, g/dl	0.86	0.83–0.89	< 0.001												
Creatinine, mg/dl	1.50	1.18–1.92	0.001												
Sodium	1.00	0.99–1.02	0.164												
INR	2.89	1.82–4.57	< 0.001												
MELD	1.16	1.11–1.21	< 0.001				1.16	1.11–1.22	< 0.001						
FIB‐4															
low	ref									ref					
intermediate	1.32	0.65–2.67	0.448							1.31	1.62–2.79	0.482			
high	2.41	1.31–4.44	0.005							2.32	1.18–4.52	0.014			
CTP															
A	ref												ref		
B	3.47	2.12–5.67	< 0.001										2.99	1.81–4.93	< 0.001
C	33.7	7.65–148	< 0.001										23.9	5.18–110	< 0.001

Patients with decompensated cirrhosis												
	Crude HR	95% CI	*p*	HR^a^	95% CI	*p*	HR^b^	95% CI	*p*	HR^d^	95% CI	*p*
Body mass index	0.97	0.92–1.02	0.172	0.98	0.92–1.03	0.389	0.97	0.92–1.03	0.313	0.96	0.90–1.02	0.185
Type 2 diabetes	1.09	0.54–2.20	0.797	0.96	0.44–2.06	0.916	1.26	0.59–2.66	0.542	1.15	0.53–2.46	0.728
AST, U/l	1.01	1.00–1.01	0.001									
ALT, U/l	1.00	0.99–1.00	0.586									
Bilirubin, mg/dl	1.35	1.16–1.57	< 0.001									
Total platelet counts, 10^9^	0.99	0.99–1.00	0.059									
Albumin, g/dl	0.94	0.90–0.98	0.004									
Creatinine, mg/dl	1.98	1.03–3.78	0.038									
Sodium	0.94	0.87–1.01	0.096									
INR	2.14	1.21–3.81	0.009									
MELD	1.12	1.06–1.19	< 0.001				1.13	1.06–1.20	< 0.001			
FIB‐4												
low	no outcomes											
intermediate	no outcomes											
high	39/61 outcomes											
CTP												
A	ref									ref		
B	2.02	0.92–4.42	0.081							2.77	1.14–6.69	0.024
C	3.61	1.47–8.88	0.005							4.04	1.56–10.5	0.004

Abbreviations: ALT, alanine transaminase; AST, aspartate transferase; CI, confidence interval; CTP, Child‐Turcotte‐Pugh; FIB‐4, fibrosis‐4 score; HR, hazard ratio; INR, international normalised ratio; MELD, model for end‐stage liver disease.

^a^ HR adjusted for age, sex, calendar year, body mass index, and type 2 diabetes.

^b^ HR adjusted for age, sex, calendar years, body mass index, type 2 diabetes, and MELD score.

^c^ HR adjusted for age, sex, calendar years, body mass index, type 2 diabetes, and FIB‐4.

^d^ HR adjusted for age, sex, calendar years, body mass index, type 2 diabetes, and CTP category.

## 4 Discussion

In this nationwide cohort study involving 2318 patients with compensated cirrhosis due to MASLD between 2001 and 2020, 55.7% developed decompensated events, underwent transplantation, or died during a median follow‐up of 1.1 years. Further, 59.7% of patients with decompensated cirrhosis due to MASLD died or underwent liver transplantation over a median follow‐up of approximately half a year. The higher progression rate was largely attributed to higher overall mortality in both groups, with decompensated events occurring in 18.2% of patients with compensated cirrhosis at an annual rate of around 8%. Higher CTP and MELD scores were associated to faster progression in both compensated and decompensated cirrhosis, whereas type 2 diabetes was linked to faster progression only in compensated cirrhosis. The large proportion of patients with decompensated cirrhosis at baseline, and the quick rate to develop outcomes, signals that patients with MASLD cirrhosis are often clinically found late in the disease process where prognosis is dismal, calling for improved means for early detection.

### 4.1 Comparison With Previous Studies

The rates of disease progression observed during the relatively short follow‐up are generally higher than previously reported from clinical studies [5, 7, 20, 21]. In a recent study including 167 patients with histology‐defined F4 fibrosis by Sanyal et al. the incidence rates were 2.7/100 PY for decompensated cirrhosis and 1.8/100 PY for all‐cause mortality, whereas our study found higher estimates of 7.9/100 PY for decompensated cirrhosis and 18.2/100 PY for all‐cause mortality [7]. Similarly, a multicenter study reported that 19.4% (48/247) of patients with advanced fibrosis or cirrhosis due to MASLD developed liver‐related complications over an average follow‐up of seven years [8]. However, these results are not directly comparable to our findings due to the inclusion of patients with stage 3 fibrosis and the inclusion of patients from prior trials with relatively healthy profiles. Our estimates align with a similar large, population‐based study by Allen et al. which reported a 33% progression rate from compensated cirrhosis to first decompensated event over four years [22]. The annual incidence (7.9/100 PY) reported in our study also aligns with the range of the results from an international cohort study including patients with advanced fibrosis due to nonalcoholic steatohepatitis, where the incidence rate for decompensated cirrhosis was 3.3/100 PY in cirrhosis with CTP A5 and 15.6/100 PY in CTP A6 [8]. In addition, rates of progression to decompensation were comparable in the clinical cohort in our study at 7.2/100 PY.

A higher incidence rate of HCC was observed in compensated cirrhosis compared to previous studies that used cohorts of histologically defined MASLD‐cirrhosis in Sweden and elsewhere, especially within the first year of cirrhosis diagnosis [23, 24]. All‐cause mortality was also higher in compensated and decompensated cirrhosis compared to previous studies [7, 8, 22]. This difference is likely due to the fact that patients diagnosed using biopsy may be on the earlier spectra of liver cirrhosis. Moreover, patients included in clinical trials or at specialised care may receive better care and thereby have better prognosis. The study population in our study was relatively old (median age 70 years) and had a high prevalence of cardiometabolic disease (88.7% had type 2 diabetes vs. approximately 70% in other studies, likely partly due to the method we used to define MASLD). Consequently, the all‐cause mortality rates, particularly deaths due to endocrinology‐related disease and extrahepatic cancer, were higher in patients with cirrhosis, in contrast to data from biopsy‐proven patients where more than 50% with MASLD‐cirrhosis died from liver disease [25]. These results are partially consistent with those from a register‐based study from Canada, which showed that non‐hepatic cancer was the main cause of death in compensated cirrhosis [26].

### 4.2 Implications

These data suggest the rate of progression to decompensated cirrhosis in patients with compensated cirrhosis due to MASLD is approximately seven incident cases per 100 person‐years. This is relevant for tailoring patient follow‐up and clinical trial design. Further, patients with type 2 diabetes may require closer follow‐up and evaluation. Mortality seems to depend largely on where in the healthcare system patients are diagnosed, with these results suggesting MASLD cirrhosis is often identified at a stage where comorbidities are frequent, and holistic care is needed to improve the prognosis. Indeed, we have recently shown that patients with MASLD in general have higher mortality rates from almost all causes compared to matched controls from the general population [27]. Additionally, there is growing interest in clinical trials in the MASLD cirrhosis area. For instance, the FGF21‐agonist efruxifermin was recently shown to have potential for cirrhosis reversal [28]. Phase III trials in this area may benefit from the natural history data presented here, but we also show the challenges in identifying patients in time for a drug to have a meaningful effect and the frequent comorbidities may hinder patient recruitment.

### 4.3 Comparison Between Register‐Based and Clinical Based Cohort

The comparisons between the register cohort and the clinical cohort are complicated by differences in patient characteristics and management across levels of care. The clinical cirrhosis cohort, being slightly younger and less likely to have type 2 diabetes (Table S2), showed lower progression rates to any severe outcome in patients with compensated cirrhosis (104.5/1000 PY) than in the register‐based cohort (243/1000 PY) (Table S3). This was driven by a lower overall mortality rate in the clinical cohort (63.1/1000 PY vs. 182.3/1000 PY). The decompensated cirrhosis rates were, however, comparable (7.1/100 PY vs. 8.2/100 PY). Patients with decompensated cirrhosis in the clinical cohort at baseline had higher liver transplantation rates and lower mortality than those in the register‐based cohort. Since it is uncommon in Sweden to offer liver transplantation to patients over 70 years old, this may explain the higher transplantation rates observed in the clinical cohort. Collectively, the differences between the cohorts indicate similar liver disease severity, but that patients diagnosed in the register‐based cohort may have been more likely diagnosed with MASLD cirrhosis incidentally during care for other diseases, explaining the high mortality rate and highlighting both the need for an earlier diagnosis of MASLD with cirrhosis and the importance of multi‐modal care for several diseases.

### 4.4 Prognostic Factors for Severe Outcomes

MELD and CTP scores were found to be predictors of prognosis in liver cirrhosis, which is well‐established across different etiologies in previous studies and confirmed in MASLD [2]. Moreover, we observed that type 2 diabetes was associated with a higher rate of decompensated cirrhosis, consistent with some studies [22, 29], suggesting stratification on diabetes status may be needed in clinical trials. Also, type 2 diabetes has been associated with faster fibrosis progression rates in MASLD and a higher overall mortality in patients with cirrhosis, highlighting a need for holistic therapy in those with both conditions [30, 31]. In patients with decompensated cirrhosis, type 2 diabetes did not appear to be associated with increased overall mortality or transplantation. This is likely due to decompensated cirrhosis being a more severe disease state, reducing the relative impact of diabetes on mortality in this vulnerable patient population.

### 4.5 Strengths and Limitations

A major strength of this study is the large patient cohort derived from a nationwide register, with follow‐up of up to 20 years and minimal loss to follow‐up. Additionally, we used validated definitions for cirrhosis and MASLD and employed a competing risk approach to accurately describe cumulative incidence of outcomes. Furthermore, the estimates were derived from different levels of healthcare to inform natural history of MASLD at various levels of care. Nevertheless, several limitations should be acknowledged. First, potential misclassification may be present in the register‐based cohort, in that patients with cirrhosis coding without explicitly having MASLD but with type 2 diabetes or obesity may be misclassified as MASLD‐cirrhosis, though this algorithm has shown a high PPV and been used in other studies [32, 33]. Second, high rates of decompensated cirrhosis and HCC occurring within the first year after diagnosis of compensated cirrhosis may reflect a delayed diagnosis of such outcomes that are likely to have been present at initial cirrhosis diagnosis, or the identification of patients at the more severe end of the compensated cirrhosis spectrum with a higher risk for severe outcomes. Our estimates should therefore be interpreted as the event rate one can expect in a clinically diagnosed patient with cirrhosis in Sweden during this period, and not as the “true” rate of progression in patients with MASLD‐cirrhosis. Unfortunately, lab data to calculate MELD or CTP scores, VCTE measurements etc. were not available in the register‐based cohort, and therefore we could not reliably determine cirrhosis severity. Furthermore, the small sample size in the clinical cirrhosis cohort may have limited our ability to detect differences between groups, especially in the decompensated stratum. Although the cumulative incidence for severe clinical events was lower after excluding patients who developed MALO within the first year, it followed a similar trend for liver‐related outcomes to that from the register‐based cohort. Although the positive predictive values for diagnoses used in this study are high, compensated cirrhosis is often asymptomatic, why patients with undiagnosed cirrhosis could not be identified. However, the clinical outcomes of decompensated events manifest suddenly and often require immediate medical intervention, why we likely captured all patients with hepatic decompensation due to MASLD. Fourth, despite being an attractive therapeutic outcome, recompensation in cirrhosis could not reliably be assessed due to the challenges in differentiating between true regression and delays in administrative coding from register‐based data. Lastly, we observed that liver‐related death was not the main cause of death in patients with cirrhosis. This should be interpreted with caution as cirrhosis frequently is not reported on death certificates even after autopsies [34], and therefore liver‐related death may be under‐reported. Cause of death is usually confirmed with clinical adjudication and that was not part of this study.

## 5 Conclusions

This large population‐based study describes the clinical characteristics and outcomes of patients with MASLD‐cirrhosis, categorised by compensation status in two cohorts. Patients identified in a nationwide register displayed similar rates of decompensated cirrhosis as those in the clinical cohort at around seven cases per 100 person‐years but experienced higher overall mortality, likely attributable to older age, more comorbidities at the time of diagnosis, and potentially different care received as compared to the clinical cohort. These results call for improved means to identify patients earlier in the disease course and may be relevant for clinical trial design in MASLD cirrhosis.

## Author Contributions

Study concept and design: Y.S., H.H., X.Z. Acquisition of data: J.V., M.E., H.H. Statistical analysis: L.W., Y.S. Analysis and interpretation of data: All. Drafting of manuscript: Y.S. Critical revision: All. Guarantor of article: H.H. All authors approved the final version of the article, including the authorship list. Writing Assistance: None.

## Funding

This study was partly funded by Merck Sharp & Dohme LLC, a subsidiary of Merck & Co. Inc., Rahway, NJ, USA.

## Conflicts of Interest

H.H.'s institutions have received research funding from Astra Zeneca, EchoSens, Gilead, Intercept, MSD, Novo Nordisk, Takeda, and Pfizer. He has served as a consultant, speaker, or on advisory boards for Astra Zeneca, Boehringer Ingelheim, Bristol Myers‐Squibb, GSK, EchoSens, Ipsen, MSD, and Novo Nordisk, and has been part of hepatic events adjudication committees for Arrowhead, Boehringer Ingelheim, KOWA, and GW Pharma. Xiao Zhang, Gail Fernandes, Mathew G Melaragno, and Samuel S. Engel are employees of Merck Sharp & Dohme LLC, a subsidiary of Merck & Co. Inc., Rahway, NJ, USA, and may own stock and/or hold stock options in Merck & Co. Inc., Rahway, NJ, USA. J.V. reports advisory boards for Novo Nordisk and lecture fees from Norgine and Gore Medical.

## Supporting information


**Data S1:** liv70487‐sup‐0001‐supinfo.docx.

## Data Availability

The data that support the findings of this study are available on request from the corresponding author. The data are not publicly available due to privacy or ethical restrictions.
