# Supplementary material for: Characteristics and Clinical Outcomes in Patients With Cirrhosis due to MASLD in Sweden
Source: Liver Int. 2025 Dec 26;46(2):e70487. doi: 10.1111/liv.70487 (PMC12741901; doi:10.1111/liv.70487)

Supplementary material

**Supplementary Table 1.** International Classification of Disease (ICD) and Anatomical Therapeutic Chemical Classification System (ATC) codes for diseases and medications

| **Diseases** | **ICD 10** |
| --- | --- |
| **Liver-related diseases** |  |
| ***MASLD*** | K76.0, K75.8 |
| ***Cirrhosis*** |  |
| *Compensated cirrhosis* |  |
| Liver cirrhosis, unspecified | K74.6 |
| Esophageal varices (not bleeding) | I85.9, I98.2 |
| Gastric varices (not bleeding) | I86.4 |
| *Decompensated cirrhosis* |  |
| Ascites | R18 without any code for heart failure (I50) or non-HCC cancer (any C, except C22.0) |
| Esophageal varices (bleeding) | I85.0, I98.3 |
| Hepatic encephalopathy | K74.6 + ATC-code A07AA11(rifaximin) within 30 days. |
| *Liver transplantation* |  |
| Diagnostic codes | Z94.4 |
| Procedure codes | JJC00, JJC10, JJC20, DJ005, DJ006, JJC30, JJC40 |
| ***Other liver disease*** |  |
| Alcohol-related cirrhosis | K70.3 |
| **Alcohol use disorder (AUD)** | F10, or codes associated with somatic consequences of alcohol: E24.4, G62.1, I42.6, K29.2, G31.2, G72.1, K85.2, K86.0, T51.0, T51.9, Y15, Y57.3, Y90, Y91, X65, Z50.2, Z71.4, Z72.1 |
| **Alcohol related live disease** | K70, AUD diagnosis+ any of the following: K74.6, R18.9, I85.0, I85.9, I98.2, I98.3, K76.7, K72.0, K72.1, K72.9, K76.6, C22.0 |
| Viral hepatitis | B16, B17, B18, B19 |
| Primary biliary cholangitis | K74.3, K74.5 |
| Primary sclerosing cholangitis | (K50 or K51) +K83.0 OR K83.0A |
| Autoimmune hepatitis | K75.4 |
| Budd-Chiari syndrome | I82.0, K76.5 |
| Alpha-1 antitrypsin-deficiency | E88.0A, E88.0B |
| Wilson’s disease | E83.0B |
| Hemochromatosis | E83.1 |
| **Other diseases** |  |
| *Cardiovascular disease* | I20-25, I60-69, I73.9 |
| Extrahepatic Cancer | C00-C97 (except C22.0) |
| Respiratory diseases | J00-J99 |
| Endocrine | E00-E90 |
| Hypertension | I10-I15 or ATC: C03AA, C07AB, C08C, C09 |
| Hyperlipidemia | E78 or ATC: C10AA |
| Type 2 diabetes | E11 or ATC: A10 |
| Depression | F30-34, F38, F39, F412 or ATC: N06AB, N06AC-N06AX |
| Renal impairment | I13.1, I13.2, I12.0, N18 |
| Hypothyroidism | E03.9 |
| COPD | J44 and age at diagnosis>=45 |
| Obesity | E65, E66 |
| **Medications** | **ATC** |
| Anti-diabetic medication | A10 |
| Metformin | A10BA02 |
| GLP1-RA | A10BJ |
| TZD | A10BG |
| SGLT-2 inhibitors | A10BK |
| Others | A10B, A10X |
| Insulin | A10A |
| Anti-hypertensive medication | C03AA, C07AB, C08C, C09 |
| ACE/ARB | C09 |
| Beta Blockers | C07AB |
| Diuretics | C03AA |
| Calcium blockers | C08C |
| Anti-Depressant |  |
| SSRI | N06AB |
| Others | N06AC-N06AX |
| Antihyperlipidemic drugs | C10AA, C10AB, C10AX |
| Statins | C10AA |
| Others | C10AB, C10AX |
| Vitamin E | A11HA03 |

Supplementary Table 2. Baseline characteristics of the clinical cirrhosis cohort (present as n (%) or median (IQR)) from the clinical cirrhosis cohort

|  | **Clinical cirrhosis cohort** | | | |
| --- | --- | --- | --- | --- |
|  | all | Compensated cirrhosis | Decompensated cirrhosis |  |
| no. of individuals | 293 | 225 | 68 |  |
| **Demographic** |  |  |  |  |
| Age at diagnosis | 67 (60-73) | 67 (59-73) | 68 (61-74) |  |
| Female | 134 (45.7) | 112 (49.8) | 22 (32.4) |  |
| **Diagnostic modalities** |  |  |  |  |
| Biopsy | 109 (37.2) | 100 (44.4) | 9 (13.2) |  |
| Imaging | 168 (57.3) | 117 (52.0)) | 51 (75.0) |  |
| Clinical diagnosis | 16 (5.5) | 8 (3.6) | 8 (11.8) |  |
| **Clinical information** |  |  |  |  |
| Body mass index | 30.0 (27.8-34.3) | 30.3 (28.7-34.3) | 30.0 (26.0-33.3) |  |
| **Comorbidity** |  |  |  |  |
| Ascites | 52 (17.7) | 0 ( 0.0) | 52 (77.8) |  |
| Varices bleeding | 19 (6.5) | 0 ( 0.0) | 19 (27.9) |  |
| Hepatic encephalopathy | 17 (5.7) | 0 ( 0.0) | 17 (25.0) |  |
| Type 2 diabetes | 201 (68.6) | 147 (65.3) | 54 (79.4) |  |
| Obesity | 143 (48.8) | 33 (48.5) | 110 (48.9) |  |
| **Lab tests** |  |  |  |  |
| AST, U/l | 52 (39-74) | 51 (39-74) | 53 (41-74) |  |
| ALT, U/l | 48 (33-76) | 52 (36-88) | 37 (26-51) |  |
| Bilirubin, mg/dl | 0.82 (0.59-1.23) | 0.70 (0.53-1.11) | 1.17 (0.82-1.87) |  |
| Total platelet counts, 10^9^ | 146 (96-205) | 157 (104-217) | 112 (69-155) |  |
| Albumin, g/dl | 3.6 (3.1-3.9) | 3.7 (3.4-3.9) | 2.8 (2.3-3.2) |  |
| Creatinine, mg/dl | 0.81 (0.68-1.01) | 0.79 (0.66-0.94) | 0.98 (0.76-1.22) |  |
| Sodium | 139 (137-141) | 140 (138-142) | 138 (135-139) |  |
| INR | 1.1(1.0-1.3) | 1.1 (1.0-1.2) | 1.3 (1.2-1.5) |  |
| **Scoring system** |  |  |  |  |
| MELD, continous | 8 (7-11) | 8 (7-10) | 11 (10-15) |  |
| CTP, continous | 6 (5-7) | 5 (5-6) | 8 (7-9) |  |
| FIB-4, continous | 3.57 (2.21-6.08) | 3.18 (2.05-5.12) | 5.95 (3.83-8.72) |  |

Abbreviations: AST: aspartate transferase ALT: alanine transaminase; INR: international normalized ratio; MELD: model for end-stage liver disease; FIB-4:fibrosis-4 score; CTP: Child-Turcotte-Pugh

**Supplementary Table 3**. Clinical outcomes in patients with cirrhosis due to MASLD from the clinical cirrhosis cohort

|  | Compensated cirrhosis | | | Decompensated cirrhosis | | |
| --- | --- | --- | --- | --- | --- | --- |
|  | No. Event/No. cases | Follow-up years (median, IQR) | IR (per 1000 py) | No. Event/No. cases | Follow-up years (median, IQR) | IR (per 1000 py) |
| **Severe clinical events** | 113/225, (50.2%) | 3.82 (2.15-6.49) | 104.5 (86.9-125.7) | 50/68, (73.5%) | 1.24 (0.50-2.61) | 393.2 (298.0-518.8) |
| Decompensated | 78/225, (34.7%) | 3.84 (2.18-6.51) | 71.5 (57.3-89.3) | Na |  |  |
| ascites | 50/225, (22.2%) | 4.75 (2.61-7.09) | 41.3 (31.3-54.5) | Na |  |  |
| encephalopathy | 42/225, (18.7%) | 4.60 (2.70-7.20) | 34.2 (25.3-46.3) | Na |  |  |
| variceal bleeding | 19/225, (8.4%) | 4.99 (3.07-7.21) | 14.5 (9.27-22.8) | Na |  |  |
| Liver transplantation | 13/225, (5.8%) | 4.92 (3.04-7.20) | 10.1 (5.88-17.4) | 17/68, (25.0%) | 1.24 (0.50-2.62) | 133.7 (83.1-215.1) |
| **HCC** | 38/225, (16.9%) | 4.79 (2.72-7.2) | 30.3 (22.1-31.7) | 7/68, (10.3%) | 1.80 (0.82-3.32) | 42.1 (20.0-88.4) |
| **Liver-related mortality** | 32/225, (14.2%) | 5.27 (3.24-7.49) | 23.4 (16.6-33.1) | 22/68, (32.3%) | 2.17 (0.96-3.77) | 111.8 (73.7-169) |
| **All-cause mortality** | 86/225, (38.2%) | 5.27 (3.24-7.49) | 63.1 (51.5-77.9) | 37/68, (54.4%) | 2.17 (0.96-3.77) | 188.2 (136.4-259.7) |

Abbreviations: MALO: major adverse liver outcomes; HCC: hepatocellular carcinoma

**Supplementary Table 4.** Cumulative incidence (%) and 95% confidence interval of the clinical outcomes in patients with cirrhosis due to MASLD from outpatient care from the register-based cohort

|  | 1 year | 5 years | 10 years | Full follow-up |
| --- | --- | --- | --- | --- |
| **Patients with compensated cirrhosis from outpatient care** | | | | |
| **Severe clinical events** | 21.4 (18.7-24.3) | 51.0 (46.7-55.2) | 71.2 (65.5-76.1) | 92.6 (77.5-97.7) |
| Decompensated cirrhosis | 9.2 (7.4-11.3) | 19.7 (16.6-23.0) | 25.8 (21.5-30.4) | 27.3 (22.3-32.5) |
| Liver transplant | 1.3 (0.7-2.3) | 3.7 (2.3-5.6) | - | 5.4 (3.4-8.1) |
| All-cause mortality | 14.4 (12.1-17.0) | 41.9 (37.7-46.1) | 64.4 (58.4-69.8) | 91.1 (77.5-96.7) |
| **Liver-related mortality** | 2.0 (1.2-3.2) | 7.9 (5.8-10.5) | 11.3 (8.2-14.8) | 14.2 (8.6-21.2) |
| **HCC** | 7.8 (6.1-9.7) | 15.5 (12.7-18.5) | - | 18.8 (15.4-22.4) |
| **Patients with decompensated cirrhosis from outpatient care** | | | | |
| **Severe clinical events** | 40.6 (36.2-44.9) | 79.3 (74.0-83.7) | 90.1 (84.3-93.9) | 95.3 (86.8-98.4) |
| Liver transplant | 3.3 (1.9-5.4) | 6.3 (3.7-9.7) | - | 6.3 (3.7-9.7) |
| All-cause mortality | 37.6 (33.3-41.9) | 74.8 (69.4-79.4) | 85.7 (79.7-90.1) | 93.2 (84.0-97.2) |
| **Liver-related mortality** | 9.1 (6.8-11.8) | 14.4 (11.1-18.2) | - | 17.4 (13.2-22.0) |
| **HCC** | 10.1 (7.7-12.9) | 15.0 (11.5-19.0) | - | 18.0 (13.5-23.0) |

Abbreviations: MALO: major adverse liver outcomes; HCC: hepatocellular carcinoma

**Supplementary Figure 1**. Flowchart of the study population from the clinical cirrhosis cohort


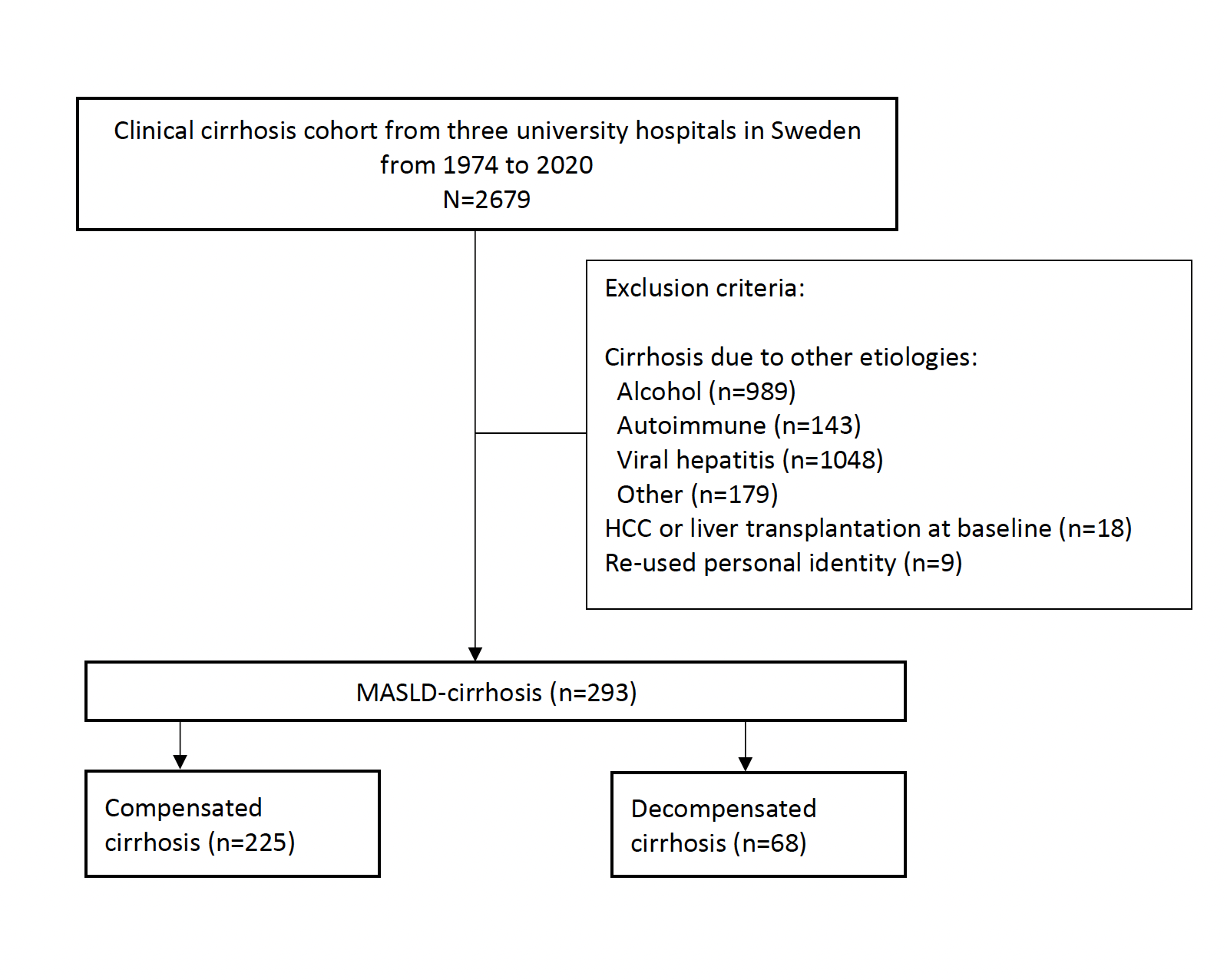

Supplement: Supplementary file 1 — Data S1: liv70487‐sup‐0001‐supinfo.docx. [file LIV-46-0-s001.docx]
